# The translocator protein gene is associated with endogenous pain modulation and the balance between glutamate and γ-aminobutyric acid in fibromyalgia and healthy subjects: a multimodal neuroimaging study

**DOI:** 10.1097/j.pain.0000000000002309

**Published:** 2021-04-09

**Authors:** Silvia Fanton, Angelica Sandström, Jeanette Tour, Diana Kadetoff, Martin Schalling, Karin B. Jensen, Rouslan Sitnikov, Isabel Ellerbrock, Eva Kosek

**Affiliations:** a Department of Clinical Neuroscience, Karolinska Insitutet, Stockholm, Sweden; b Department of Neuroradiology, Karolinska University Hospital, Stockholm, Sweden; c Stockholm Spine Center, Löwenströmska Hospital, Upplands Väsby, Sweden; d Department of Molecular Medicine and Surgery, Karolinska Institutet, Stockholm, Sweden,; e Center for Molecular Medicine, Karolinska University Hospital, Stockholm, Sweden; f MRI Research Center, Karolinska University Hospital, Stockholm, Sweden; g Department of Surgical Sciences, Uppsala University, Uppsala, Sweden

**Keywords:** Fibromyalgia, TSPO, Genetic polymorphism, Endogenous pain modulation, MRS, Glutamate, GABA, rACC, Thalamus, fMRI

## Abstract

A cerebral upregulation of the translocator protein (TSPO), a biomarker of glial activation, has been reported in fibromyalgia subjects (FMS). The TSPO binding affinity is genetically regulated by the Ala147Thr polymorphism in the TSPO gene (*rs6971*) and allows for a subject classification into high affinity binders (HABs) and mixed/low affinity binders (MLABs). The aim of the present multimodal neuroimaging study was to examine the associations of the TSPO polymorphism with: (1) conditioned pain modulation, (2) expectancy-modulated pain processing assessed during functional magnetic resonance imaging, and (3) the concentration and balance of glutamate and γ-aminobutyric acid in the rostral anterior cingulate cortex and thalamus using proton magnetic resonance spectroscopy in FMS (n = 83) and healthy controls (n = 43). The influence of TSPO on endogenous pain modulation presented in the form of TSPO HABs, as opposed to MLABs, displaying less efficient descending pain inhibition and expectancy-induced reduction of pain. Translocator protein HABs in both groups (FM and healthy controls) were found to have higher thalamic glutamate concentrations and exhibit a pattern of positive correlations between glutamate and γ-aminobutyric acid in the rostral anterior cingulate cortex, not seen in MLABs. Altogether, our findings point to TSPO-related mechanisms being HAB-dependent, brain region-specific, and non–FM-specific, although in FMS the disadvantage of an aberrant pain regulation combined with an HAB genetic set-up might hamper pain modulation more strongly. Our results provide evidence for an important role of TSPO in pain regulation and brain metabolism, thereby supporting the ongoing drug development targeting TSPO-associated mechanisms for pain relief.

## Abstract 2

Supplemental Digital Content is Available in the Text.

In subjects with fibromyalgia and healthy subjects, the genetically inferred translocator protein high-affinity binding variant was associated with brain region-specific metabolic patterns and reduced top-down pain modulation.

## 1. Introduction

Fibromyalgia (FM) is a nociplastic pain condition^36^ characterized by widespread musculoskeletal pain and affecting 2% to 4% of the general population.^23,63^ Although the etiology is not entirely understood, several central nervous system aberrations have been documented,^56^ including (1) deficient descending pain modulation, (2) altered brain metabolism, and (3) neuroinflammation.

Evidence for a dysfunctional top-down regulatory system comes from reports of deficient conditioned pain modulation (CPM)^37,46^ and from studies using functional magnetic resonance imaging (fMRI) documenting reduced activation and diminished functional connectivity within the brain's inhibitory network. Specifically, during evoked pain, FM subjects (FMS) have been shown to fail to activate the thalamus and a primary link in the descending pain inhibitory system, ie, the rostral anterior cingulate cortex (rACC), and exhibited reduced functional connectivity between rACC and brainstem.^28,29^

Furthermore, results from proton magnetic resonance spectroscopy (^1^H-MRS) demonstrated a disequilibrium of numerous metabolites in the brain of FMS, including the 2 major neurotransmitters: excitatory glutamate^11–13,19–21,59^ and inhibitory γ-aminobutyric acid (GABA).^15^ Despite their physiological interactions^33^ and the potential pathophysiological consequences of an altered balance between excitation and inhibition, to our knowledge, no previous FM studies examined their potentially interacting cerebral concentrations.

Finally, neuroinflammation has been reported in FM as elevated concentrations of interleukin-8 and fractalkine in the cerebrospinal fluid (CSF)^2,32,35^ and upregulated expression of a biomarker of glial activation, ie, the translocator protein (TSPO), in several cortical regions, including the anterior cingulate cortex.^1^ Given that TSPO expression is upregulated in activated glia,^39,54^ TSPO positron emission tomography radioligands, such as ^11^C-PBR28, have been largely used to investigate neuroinflammatory disorders.^9,62^

The binding affinity of ^11^C-PBR28 to TSPO is genetically regulated by the Ala147Thr polymorphism (*rs6971*) in the TSPO gene.^47^ Although little is known regarding the physiological importance of this functional polymorphism, FMS who are genetically inferred TSPO high affinity binders (HABs) compared to mixed/low affinity binders (MLABs) showed heightened symptom severity and increased pain-related functional connectivity in the frontoparietal network,^38^ a network involved in expectancy-induced pain modulation.^34,60^ Consequently, it would be plausible to assume that TSPO-related mechanisms in FM influence the anticipatory, expectancy-induced components of cerebral pain processing. Translocator protein controls the rate limiting steps in neurosteroid synthesis,^8,10^ and neurosteroids exert modulatory effects on GABA-A receptors.^45^ Furthermore, as glia plays a crucial role in glutamate metabolism^26^ and TSPO is upregulated in activated glia,^39,54^ TSPO might impact glutamate signaling.

The aim of the present multimodal neuroimaging study was to explore the associations between the TSPO polymorphic variants and CPM, expectancy-induced pain modulation, cerebral pain processing (fMRI), and the concentrations of glutamate and GABA (^1^H-MRS) in regions associated with altered pain inhibition in FMS. Based on our previous findings,^28,29,38,55^ we hypothesized that FMS with TSPO HAB have more severe pain, reduced CPM, an expectancy bias towards pain-related threats, and decreased pain-related activation in pain modulatory areas (rACC and thalamus) compared to MLABs. The analysis of MRS data with respect to TSPO should be considered as exploratory.

## 2. Materials and methods

### 2.1. Study participants

A total of 84 female FMS and 43 age-balanced healthy controls (HC) (n = 127, age range: 29-60 years) was recruited in the study. Data from one FMS were excluded from the analyses because of the absence of genotype determination (refer to section 2.2.1.2). The final dataset consisted of 83 FMS and 43 HC (n = 126). Participants were recruited by advertisement in the daily press. All FMS underwent a screening by a specialist in rehabilitation medicine and pain relief so as to ensure compliance with inclusion and exclusion criteria. Inclusion criteria for FMS comprised female sex, due to an extensively reported prevalence of FM in women,^3,6^ right handedness, working age (20-60 years), as well as meeting the FM classification criteria ACR-1990 and ACR-2011. Exclusion criteria for FMS were rheumatic or autoimmune diseases, severe psychiatric disorders requiring treatments for depression or anxiety, severe somatic diseases (neurological, cardiovascular, etc.), other dominant pain syndromes than FM, previous heart or brain surgery, substance abuse, medication with anticonvulsants or antidepressants, self-reported claustrophobia, inability to refrain from hypnotics, nonsteroidal anti-inflammatory drugs, or analgesics prior to study participation, specifically 48 hours before the first visit and 72 hours before the second visit, ie, the neuroimaging session, hypertension (>160/90 mmHg), obesity (body mass index > 35), smoking (>5 cigarettes/day), magnetic implants, pregnancy, and inability to understand and speak Swedish. The HC were screened by a telephone interview. Healthy controls were right-handed women, free from FMS exclusion criteria stated above and, additionally, free from any chronic pain conditions.

All participants gave written informed consent in accordance with the Declaration of Helsinki and were compensated for their time. The study was approved by the local ethical review board (ethics permit: 2014/1604-31/1).

### 2.2. Procedure

The current study forms part of a larger project (refer to study plan https://osf.io/8zqak). While the effects of pain conditioning in FMS and HC are described in detail in a previous publication,^55^ this article explores TSPO-related effects.

Data were collected in 2 sessions on 2 subsequent days. Day 1 consisted of (1) the administration of a set of validated questionnaires, (2) the collection of saliva samples for genotyping, (3) the calibration of individual pressures to be used in the scanner on day 2, (4) the conditioning phase of an instructed pressure pain conditioning paradigm, (5) the assessment of pressure pain thresholds (PPTs) via pressure algometry on 8 different body sites, and (6) the assessment of CPM. Day 2, the multimodal neuroimaging session, consisted of (1) single-voxel ^1^H-MRS performed in the right rACC and bilateral thalamus and (2) task-based fMRI, in which subjects underwent another conditioning phase and the testing phase of the instructed pressure pain conditioning paradigm.

#### 2.2.1. Day 1

##### 2.2.1.1. Questionnaires

Fibromyalgia subjects completed the Fibromyalgia Impact Questionnaire (FIQ),^5^ a 20-item questionnaire assessing FM-related symptoms and disability. The FIQ yields a total score between 0 and 100, with higher scores indicating poorer health. All subjects were further administered with the visual analogue scale (VAS) for pain intensity ratings, the Short Form-36 (SF-36) health survey,^7^ the Hospitalized Anxiety and Depression Scale (HADS),^64^ and the Pain Catastrophizing Scale (PCS).^57^ Visual analogue scale ratings were provided using a 0–100 mm scale, ranging from “no pain” to “worst imaginable pain,” with subjects rating their current pain (VAS current) as well as the average pain intensity experienced during the past week (VAS past week). The bodily pain (SF-36BP) is a subscale of the SF-36, assessing pain severity and pain interference with working activities, including housework, over a longer period (4 weeks).^24^ On a 0 to 100 scale (converted from raw scores), lower scores of the SF-36BP indicate more pain symptoms. The HADS is a psychometric questionnaire for nonpsychiatric patients. In HADS, 2 subscales assess anxiety and depression, HAD-A and HAD-D, respectively, each rated on a 21-point scale (0: no anxiety or depression and 21: maximal anxiety or depression). The PCS includes 13 items, each rated on a 5-point scale (0: not at all and 4: all the time), assessing the 3 subscales rumination, magnification, and helplessness. Higher PCS scores correspond to more pain-related catastrophizing.

##### 2.2.1.2. Translocator protein (rs6971) genotyping

Saliva samples were collected using Oragene kits (OG-500). The genotyping procedure was performed using TaqMan SNP genotyping assays as well as ABI 7900 HT instrument (Applied Biosystems (ABI), Foster City, CA). Polymerase chain reactions (PCR), with 5 µL of total volume, were performed in 384-well plates containing 2.5 µL Universal Master Mix and 5 ng dried-down genomic DNA per well. The PCR amplification protocol comprised 2 holds, 50°C for 2 minutes and denaturation for 10 minutes at 95°C, and was then followed by 45 cycles at 92°C for 15 seconds and 60°C for 1 minute.^38^

##### 2.2.1.3. Suprathreshold pressure pain (P10, P50) calibration

Stimulus intensity was individually calibrated to match subjective pain ratings of 10 mm and 50 mm (hereinafter referred to as P10 and P50) on a 100-mm VAS. A pressure stimulus was exerted on the participants' left calf by means of a 13 × 85-cm cuff connected to a rapid cuff inflation system (E20/AG101, Hokanson, WA). Cuff pressure algometry was used as it targets deep tissue nociceptors in a more efficacious and ecologically valid way to induce deep tissue pain similar to FM pain. In previous studies, a cuff inflator was successfully used on FMS.^41^ During pain calibration, participants were administered 5-second stimulations in an ascending series starting from 25 mmHg, with steps increasing in intensity by 25 mmHg, so as to determine the first pressure eliciting pain (first VAS > 0 mm) and the stimulation maximum (first VAS > 60 mm). Subsequently, participants were presented with 2 series of 5 randomized stimuli each, one series to determine the individual representation of P10 (VAS 10 mm) and the other one of P50 (VAS 50 mm). While the former used the first pressure eliciting pain as starting point and up to −2 and +2 steps of 25 mmHg each, the latter used the stimulation maximum as starting point and up to −4 steps of 25 mmHg each. In case the first pressure eliciting pain was < 100 mmHg, the randomized series to determine P10 was presented with increasing steps of 10 mmHg instead of 25 mmHg.

##### 2.2.1.4. First conditioning phase of the instructed pressure pain conditioning paradigm

Participants underwent the first conditioning phase of the instructed pain conditioning paradigm in front of a computer in a behavioral laboratory to associate color cues with pressure stimuli of different intensities. Here, participants were explicitly instructed and subsequently trained to pair a green circle with their calibrated P10 (P10green) and a red circle with P50 (P50red). The order was pseudorandomized (10 repetitions of P10green and 10 repetitions of P50red), and participants were instructed to rate their perceived pain intensity on a computerized VAS after each pressure stimulus.

##### 2.2.1.5. Assessment of pressure pain thresholds

Pressure pain thresholds, an indicator of pain sensitivity, were assessed using a hand-held pressure algometer (Somedic Sales AB, Hörby, Sweden), with a hard rubber probe of 1 cm^2^ applied with a 90° angle and a steadily increasing pressure rate of approximately 50 kPa/second. All participants were familiarized with the algometer prior to testing. Pressure pain thresholds were recorded when participants pressed a button, signaling that they perceived the pressure as slightly painful. Pressure pain thresholds were assessed bilaterally at 4 anatomical sites: the supraspinatus muscle, lateral epicondyle (elbow), gluteus muscle, and medial fat pad (knee). The average PPT (PPT_mean_) for all 8 assessments was calculated for each subject and later used in the analysis.

##### 2.2.1.6. Assessment of conditioned pain modulation

Conditioned pain modulation was determined with PPTs as test stimuli and ischemic pain as conditioning stimulus (the Tourniquet test). In assessing PPTs, the hand-held pressure algometer was used and the handling procedure was performed as described above, with the difference that the quadriceps femoris muscle (right thigh) was used as only anatomical target. The continuous ischemic pain was induced using a 7.5-cm wide blood cuff pressure gauge placed on the participants' upper left arm.

Participants were in a comfortable half-seated position. Before starting with the Tourniquet test, PPTs were assessed twice (first and second PPT baseline). Then, the experimenter kept the participants' left arm raised for 1 minute, in order to drain the venous blood. At this point, the cuff was adjusted and inflated to 200 mmHg and the arm was placed back to the horizontal position. In order to induce ischemic pain in the participants' left arm, participants were instructed to lift a 1-kg weight by extending the wrist. Participants were asked to rate, once every few extensions, their perceived pain intensity on a VAS (0-100 mm) scale. As soon as the VAS rating exceeded 50 mm, the lifting was ended. At this stage, the experimenter began assessing the PPTs on the participants' right thigh by means of the pressure algometer. Pressure pain thresholds were assessed continuously, with at least 10-second intervals between assessments for a duration of 4 minutes or until the participants decided to end the procedure (end PPT value). After 5-minute rest, PPTs were assessed again twice.

#### 2.2.2. Day 2: multimodal neuroimaging

MR scanning was performed on a 3T whole-body scanner (MR750, General Electric, Milwaukee, Wisconsin) using an 8-channel head receiver array (InVivo Inc).

##### 2.2.2.1. Single-voxel proton magnetic resonance spectroscopy (^1^H-MRS) data acquisition

The voxel position was verified by 3-plane localizer images performed before every MRS scan. Gradient echo shimming, frequency, and water suppression adjustments were automatically accomplished before each data acquisition. To ensure comparisons of our results with other studies and particularly for clinical subjects, the conventional PRESS (point resolved spectroscopy) was chosen with the following parameters: TR/TE/TE1 = 2000/40/19 milliseconds (ms), spectral bandwidth 5 kHz, 4096 time-domain data points, and water suppression by 3 CHESS (chemical shift selected suppression) prepulses. To enhance the voxel definition, 6 very sharp outer volume suppression radio frequency pulses surrounding voxel were applied. The voxel volume was 5.4 mL and 12 mL for rACC and thalamus, respectively. Both voxels acquired with 128 as number of averages and eight-step phase cycle resulted in experimental time of 5 minutes for each voxel.

##### 2.2.2.2. Functional magnetic resonance imaging data acquisition

A total of 320 volumes each comprised of 42 axial slices (slice thickness 3 mm and 0.5 mm gap) was acquired using a T2*-sensitive gradient echo-planar imaging sequence (TR 2 seconds; TE 30 ms; flip angle 70°; field of view 220 × 220 mm, 72 × 72-mm matrix; and 3 × 3-mm in-plane resolution). The first 5 functional volumes were discarded to account for T1 equilibrium effects. For spatial normalization, a high-resolution T1-weighted structural scan (3D IR-SPGR “BRAVO,” TI 450 ms, FA/TR 12°/7.1 ms, voxel size 1 × 1 × 1 mm, 176 slices) was also obtained.

During fMRI, participants performed the second phase of the instructed pressure pain conditioning paradigm, followed by the test phase. First, participants repeated the conditioning phase with 10 repetitions of P10green and 10 repetitions of P50red presented in a pseudorandomized order. After a short break, participants underwent the testing phase, which probed whether cue associations led to expectancy modulation of pain. As a reminder boost, the first 4 stimulations of the testing phase were presented identically as in the conditioning phase, with 2 repetitions of P10green and 2 repetitions of P50red. Subsequently, participants were exposed to a new pressure stimulus (P30), which was identically delivered after both colors, ie, red and green cues. P30 was determined by averaging P10 and P50, resulting in an individual midintensity pressure pain stimulus (P30 = (P10 + P50)/2). P30 was presented in a pseudorandomized order with 10 repetitions of P30-green (P30green) and 10 repetitions of P30-red (P30red) for a duration of 5 seconds before being prompted to rate pain intensity on a 0–100 VAS (8 seconds). All stimuli onsets were jittered over the course of the paradigm. The duration of each part was approximately 11 minutes. Here, only results from the experimental testing phase of cue-stimulus associations are reported.

### 2.3. Statistics

#### 2.3.1. Clinical parameters and behavioral data

All analyses of subject characteristics (n = 126), clinical parameters, and behavioral data were performed using R version 1.1.463.^53^ Statistical significance was set at the conventional *P* < 0.05. Data from individuals with genetically inferred TSPO LAB (n = 9) and TSPO MAB (n = 43) were combined and treated unitedly and are hereinafter referred to as MLABs (n = 52).^40^ The remaining subjects (n = 74) in the cohort were individuals with the genetically inferred TSPO HAB variant.

##### 2.3.1.1. Subject characteristics and effects of translocator protein polymorphism on clinical parameters

Differences in age between FMS and HC as well as between FM HABs and MLABs were analyzed through 2 one-way analysis of variance (ANOVA) tests. Analyses of the effects of TSPO polymorphism and group on VAS current, VAS past week, SF-36BP, HADS, PCS, and PPT_mean_ were performed by using, separately for each measure, a two-way ANOVA with 2 factors, each with 2 levels: TSPO polymorphism (HAB, MLAB) and group (FM, HC). All the above-mentioned parameters, in addition to FM duration, tender points, and FIQ, were further compared between FMS with the different genetically inferred variants of TSPO (HAB, MLAB) by means of a one-way ANOVA. The distribution of the genetic variants of TSPO in the groups (FM, HC) was assessed by performing a chi-squared (χ^2^) test.

##### 2.3.1.2. The conditioned pain modulation score

A CPM score was calculated for each participant as (end PPT value – first PPT baseline)/first PPT baseline, thus controlling for baseline variability.^40,58^ As outcomes, a positive score represented inhibition, a negative score facilitation, and zero corresponded to no pain modulation. The effects of the TSPO polymorphism and group on pain modulation were tested by a two-way ANOVA with 2 factors, each with 2 levels: TSPO polymorphism (HAB, MLAB) and group (FM, HC).

##### 2.3.1.3. Sensitivity to suprathreshold pressure pain (P10, P50)

Differences in individually calibrated input pressures (mmHg) were analyzed by performing a mixed ANOVA with 3 factors, each with 2 levels: pressure level (P10, P50), group (HC, FM), and TSPO polymorphism (HAB, MLAB). Pressure level was a within-subject variable, whereas TSPO polymorphism and group were between-subject variables.

##### 2.3.1.4. Experimental testing phase of the instructed pressure pain conditioning paradigm

A linear mixed-effects model was performed using the *nlme* package^51^ to analyze the effects of TSPO polymorphism, group, time, and cue color on subjective pain ratings acquired during the testing phase of the paradigm. All possible two-way interactions among the variables TSPO polymorphism (HAB, MLAB), group (FM, HC), cue color (green, red), and time (10 repetitions per cue color) were entered into the model as fixed effects. Random intercepts for subjects and by-subject-over-time random slopes were entered into the model to account for, respectively, the variability among subjects at baseline and the individual variability in the effect over time. The model was adjusted for the Restricted Maximum Likelihood Estimation, and an autocorrelation structure of order 1 (corAR1) was introduced to account for the intrasubject dependency stemming from having multiple measures per subject. For all effects, the 95% confidence interval is reported. In order to further explore the results obtained from the main model, 2 repeated measures ANOVAs were used testing for the effects of TSPO polymorphism, group, and time on pain ratings separately for the green and red conditions. Follow-up analyses were evaluated at *P* = 0.025 in order to correct for multiple comparisons.

#### 2.3.2. Multimodal neuroimaging data

##### 2.3.2.1. Single-voxel ^1^H-MRS

LCModel software (version 6.3-1K, s-provencher.com) was used for MRS data quantification. Before quantification, MRS data were preprocessed in MATLAB (The MathWorks, Natick, MA). The preprocessing included the S/N2-weighted MRS signal coil combining frequency and phase correction for every trace before final coherent averaging of the elementary MRS traces for each voxel. The basis set required for LCModel was simulated by quantum mechanical density matrix formalism in MATLAB using the actual timing parameters used in PRESS pulse sequence and the chemical shifts and J-coupling constants published and available elsewhere.^17,18^ The basis set included the following metabolites: aspartate, glutamate, glutamine, GABA, N-acetyl aspartate, myo-inositol and scyllo-inositol, taurine, ascorbate, glucose, creatine and phosphocreatine, choline and glycerophosphorylcholine, N-acetylaspartate-glutamate, glutathione, alanine, lactate, ethanolamine, and phosphorylethanolamine. The basis set was calibrated using MRS phantom (BRAINO + GABA, GE Healthcare). The MRS data were quantified using the ratio to (1) total creatine (7-mM assumed value, relative) and (2) total voxel water concentration (absolute). To estimate the endogenous water concentration, the MRS voxel mask was coregistered with a 3D T1-weighted image in native space that was segmented in 3 tissue types (gray matter, white matter, and CSF) in FSL (FMRIB Software Library) version 5. The obtained tissue volumes were then masked by the voxel and partial volume estimates for each tissue type that was used to correct the total water concentration.

All the hereinafter reported analyses were performed separately for rACC and thalamus datasets using the absolute and relative concentrations of glutamate and GABA.

The effects of TSPO polymorphism and group on metabolite concentrations were tested by performing two-way ANOVAs, separately for glutamate and GABA concentrations, with 2 factors, each with 2 levels: TSPO polymorphism (HAB, MLAB) and group (FM, HC).

In order to determine correlations between brain metabolites, nonparametric Spearman correlations were performed between glutamate and GABA concentrations across subjects, separately for groups (FM and HC), TSPO genotypes (HAB and MLAB), and their combinations (FM HAB, FM MLAB, HC HAB, and HC MLAB).

Similarly, Spearman correlations were performed to assess the relationship between concentrations of glutamate, GABA, and pain modulation in subgroups of groups and TSPO genotypes. Specifically, correlations were performed between glutamate and CPM score as well as between GABA and CPM score.

##### 2.3.2.2. Functional magnetic resonance imaging

First, anatomical and functional scans were manually reoriented to the anterior commissure. Preprocessing included spatial realignment to the average image, coregistration of the structural image to the mean functional data, normalization into the Montreal Neurological Institute stereotactic standard space and smoothing with a 6-mm full-width at half-maximum isotropic Gaussian kernel using the statistical parametric mapping software package (SPM12, Wellcome Trust Centre for Neuroimaging, London, United Kingdom) running under MATLAB. Eleven participants were excluded because of technical issues or dropouts resulting in incomplete MRI data (n = 9), closing their eyes during cue presentation (n = 1), or structural brain anomalies (n = 1). Framewise displacement (FD) was used to assess head movement from one frame to another by determining the derivatives' sum of absolute values of the 6 realignment parameters.^52^ As a result, 3 subjects were excluded from further analyses due to excessive head motion (FD > 0.5 in ≥ 15% of the images). No differences in FD between FMS and HC were observed (Wilcoxon rank sum test, Z = 1.2, *P* = 0.21). Data of 102 participants (FMS = 68, HC = 34) were included in the final fMRI analyses.

The general linear model approach as implemented in SPM12-7219 was used for data analysis. Further processing on the individual subject level included temporal high-pass filtering (cut-off 128 seconds) and correction for temporal autocorrelations using first-order autoregressive modelling. The individual first-level models estimated for each subject response for 2 cue/anticipation phases (red cue preceding P30/green cue preceding P30, 2 seconds cue plus a period of 2-6 seconds delay before stimulus onset), 2 pressure stimulations (P30 stimulus after green cue [P30green]/P30 stimulus after red cue [P30red], 5 seconds), and the rating period (8 seconds) after each stimulus. Epochs between trials and between trial components not specifically modeled (approximately 20 seconds per trial) were used as the implicit baseline. Regressors of interest were convolved with a canonical hemodynamic response function (HRF). Six realignment-derived motion parameters were included as regressors of no interest. Single-subject contrast images from individual first-level models were used in random-effects analyses to test for TSPO effects.

##### 2.3.2.3. Region of interest based functional magnetic resonance imaging and single-voxel ^1^H-MRS

**Figure 1.**
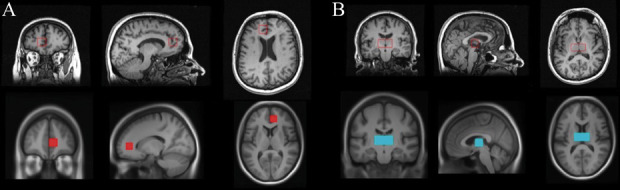
(A) Right rACC and (B) bilateral thalamus. Top row: radiological display convention of MRS single-voxel placement. Bottom row: neurological display convention of ROI positioning. MRS, magnetic resonance spectroscopy; ROI, region of interest; rACC, rostral anterior cingulate cortex.

Pain-evoked single-subject blood oxygen level-dependent (BOLD) signal extracted from thalamus and rACC were tested for possible correlations with glutamate and GABA concentrations in the same brain regions. Regions of interest in the right rACC (Fig. 1A) and bilateral thalamus (Fig. 1B) were manually drawn to correspond to the single-voxel region of interest (ROIs) used in MRS. The individual BOLD signal for the effect of painful pressure stimulation for all P30 compared to the implicit baseline was extracted from individual first-level analyses from the specified ROIs. Blood oxygen level-dependent signal was averaged over all voxels within each ROI to decrease noise. The drawing of ROIs and parameter extraction thereof were performed using the MarsBar Region of Interest Toolbox (http://marsbar.sourceforge.net). This approach was chosen to best link ROI fMRI and MRS data with each approach providing one single value (ie, BOLD signal, glutamate, and GABA) per subject averaged over each ROI (Figs. 1A and B).

Spearman correlations were then performed to test for associations between glutamate and pain-evoked BOLD signal as well as between GABA and pain-evoked BOLD signal across subjects, separately for groups (FM, HC) and TSPO genotypes (HAB, MLAB).

All the hereinbefore reported analyses were performed separately for rACC and thalamus datasets using the relative and absolute concentrations of glutamate and GABA.

In addition, rACC and thalamus masks based on MRS ROIs were combined into one mask and subjected to a ROI fMRI analysis. Specifically, two-sample *t* tests were performed to test for (1) group differences and (2) TSPO polymorphism differences in the predetermined ROIs within SPM with group as covariate in the latter. Based on behavioral results and observed TSPO effects in P30green but not in P30red, tests were performed separately for the 2 conditions. For the ROI fMRI analyses, the statistical threshold was set to *P* < 0.001.

##### 2.3.2.4. Whole-brain functional magnetic resonance imaging

In an additional analysis, we used an exploratory approach to determine whether regions outside of the predetermined regions, ie, rACC and thalamus, displayed TSPO polymorphism differences. These whole-brain analyses aimed at exploring cerebral differences in the processing of expectancy-modulated pain between TSPO genotypes and their interaction with processing of evoked pain based on cue color.

In order to link pain ratings to neural activity, additional first-level models using parametric modulation were performed, where the amplitude of the pain-related neural response was estimated using individual pressure pain ratings. The purpose of this approach was to link reported perception, ie, individual VAS scores, to neural response across stimulus repetitions of the same condition, ie, P30green or P30red. The parametric modulator was convolved with the HRF to create a regressor that represents response modulated by individual VAS scores for each stimulus. Single-subject contrast images from individual first-level models were used in random-effects analyses on the group level.

A one-sample *t* test was performed to investigate the main effect of painful stimulation (pooled across all presentations of P30). Additional tests were based on observed effects in pain ratings obtained during the testing phase of the pressure pain conditioning paradigm, specifically, to explore the interaction between TSPO and cue color. Two-sample *t*-tests were performed to test for (1) TSPO polymorphism differences during evoked pain for each cue color (P30red and P30green) and (2) TSPO polymorphism effects associated with differences in subjective pain intensity ratings (parametric modulator) during evoked pain. The factor “group” as a regressor of no interest was included in all tests exploring TSPO differences. For tests targeting brain regions outside of a priori ROIs, ie, rACC and thalamus, initial statistical parametric images were thresholded at *P* < 0.001 and a cluster threshold of *P* < 0.05 (family-wise error corrected) was applied.

## 3. Results

### 3.1. Behavioral results

#### 3.1.1. Subject characteristics and effects of translocator protein polymorphism on symptom severity

Subject characteristics and effects of group and TSPO on clinical parameters in FMS are reported in Table 1. As expected, individuals with FM, when compared to HC, provided higher ratings of pain, anxiety, depression, and catastrophizing as well as had lower PPTs. However, no main effects of TSPO polymorphism nor significant interactions between TSPO polymorphism and group were found. In addition, in the FM group, no significant effects of TSPO on any of these parameters were shown. The distribution of the genetic variants of TSPO (HAB, MLAB) did not differ between groups (χ^2^ = 1.10, *P* = 0.2932).

**Table 1:** Subject characteristics and effects of group and (FM) TSPO on clinical parameters.

	FM (n = 83) M (SD, min, max)	HC (n = 43) M (SD, min, max)	GROUP *P*	TSPO *P*	GROUP × TSPO *P*	FM HAB (n = 52) M (SD, min, max)	FM MLAB (n = 31) M (SD, min, max)	FM TSPO *P*
Age	47.3 (7.8)	48.1 (7.6)	0.5545	NA	NA	47.3 (7.9)	47.2 (7.7)	0.9635
FM duration	121.3 (87.6, 11, 408)	NA	NA	NA	NA	131.7 (91.0, 11, 408)	103.2 (79.6, 11, 288)	0.1561
Tender points	16.4 (1.8, 11, 18)	NA	NA	NA	NA	16.6 (1.9, 11, 18)	16.2 (1.8, 11, 18)	0.4629
FIQ	63.5 (16.4, 13, 95)	NA	NA	NA	NA	64.9 (14.9, 29, 95)	61.0 (18.7, 13, 91)	0.2933
VAS current	53.5 (22.1, 6, 99)	2.2 (3.2, 0, 14)	<0.001	0.5481	0.7202	54.5 (22.2, 6, 99)	51.6 (22.3, 9, 88)	0.5676
VAS past week	58.2 (21.5, 15, 100)	4.3 (5.8, 0, 26)	<0.001	0.6438	0.4503	59.4 (22.8, 15, 100)	56.1 (19.2, 22, 93)	0.4955
SF-36BP	31.0 (14.5, 0, 61)	89.0 (12.4, 51, 100)	<0.001	0.9101	0.3446	30.2 (14.7, 0, 61)	32.3 (14.1, 0, 61)	0.5322
HAD-A	7.8 (4.3, 0, 21)	3.1 (2.9, 0, 21)	<0.001	0.3464	0.9503	7.6 (4.1, 0, 19)	8.3 (4.6, 0, 21)	0.4742
HAD-D	7.4 (4.1, 0, 18)	1.1 (1.5, 0, 5)	<0.001	0.2627	0.9564	7.1 (3.7, 0, 16)	7.8 (4.7, 1, 18)	0.4344
PCS	18.1 (11.0, 0, 48)	4.7 (7.0, 0, 35)	<0.001	0.2698	0.5761	17.0 (10.8, 0, 48)	19.8 (11.2, 2, 46)	0.2757
PPT_mean_	151.4 (62.5, 39, 333)	317.2 (108.1, 77, 633)	<0.001	0.4069	0.8274	147.6 (59.6, 41, 313)	157.5 (67.5, 39, 333)	0.4881

Reported numbers are *P* values as well as means (M) with standard deviations (SD), minimum (min) and maximum (max) in parentheses.

FIQ, Fibromyalgia Impact Questionnaire; FM, fibromyalgia; HAB, high affinity binders; HAD-A, Hospital Anxiety and Depression Scale (anxiety score); HAD-D, Hospital Anxiety and Depression Scale (depression score); HC, healthy controls; MLAB, mixed/low affinity binders; NA, not applicable; PCS, Pain Catastrophizing Scale; PPT_mean_, pressure pain threshold mean; SF-36BP, Short Form-36 bodily pain score; TSPO, translocator protein; VAS, visual analogue scale.

#### 3.1.2. Effects of translocator protein polymorphism on conditioned pain modulation

**Figure 2.**
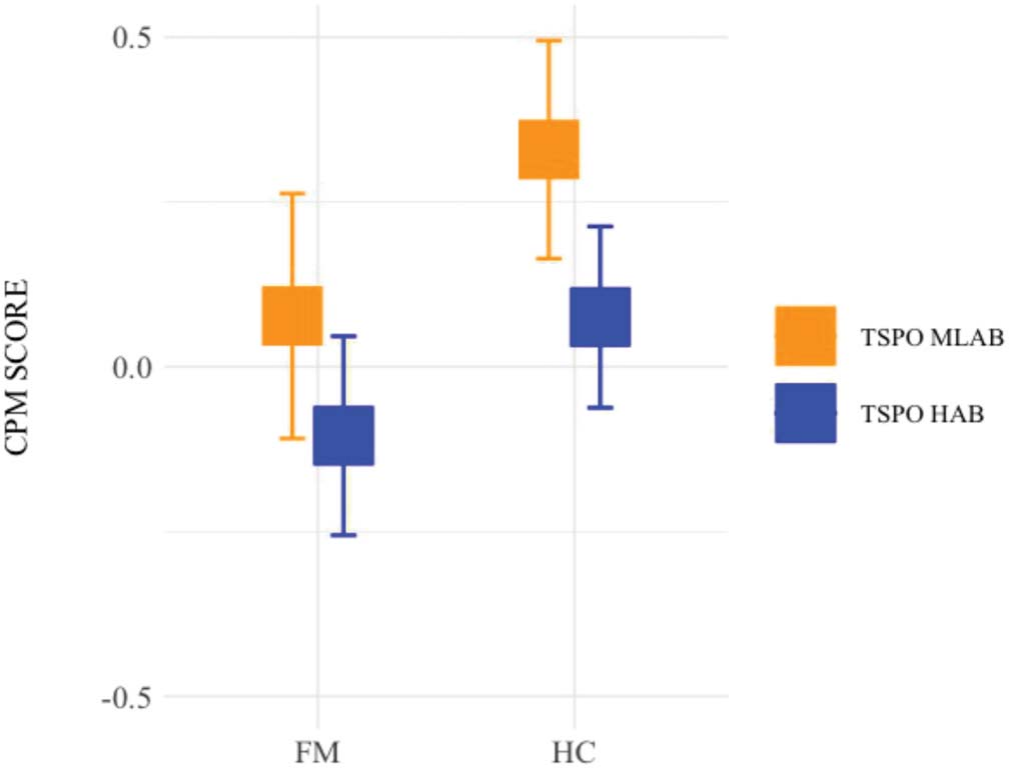
CPM score per group (FM, HC) by TSPO polymorphism (HAB, MLAB). Error bars represent the lower and the upper 95% confidence intervals. Squares represent the mean of the data. CPM, conditioned pain modulation; FM, fibromyalgia; HAB, high affinity binders; HC, healthy controls; MLAB, mixed/low affinity binders; TSPO, translocator protein; FM MLAB = 30; FM HAB = 51; HC MLAB = 21; HC HAB = 21.

The analysis of the CPM score yielded a main effect of group (F(1,119) = 7.26, *P* = 0.0081; Fig. 2), with FMS displaying decreased pain modulation compared to HC. In addition, a main effect of TSPO polymorphism was found (F(1,119) = 5.79, *P* = 0.0177; Fig. 2), with TSPO HABs displaying a reduced pain modulation compared to TSPO MLABs. No statistically significant interaction between TSPO polymorphism and group arose from the analysis (*P* = 0.6877), indicating that TSPO polymorphism influenced descending pain modulation in FMS and HC alike.

#### 3.1.3. Effects of translocator protein polymorphism on sensitivity to suprathreshold pressure pain (P10, P50)

**Figure 3.**
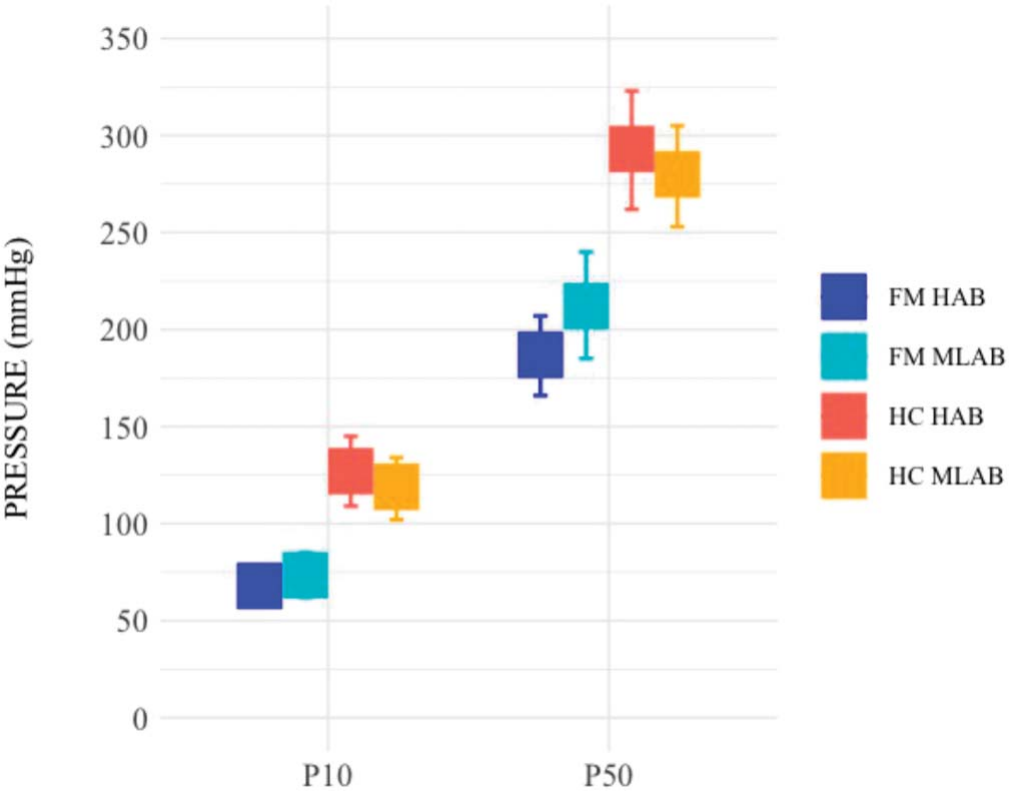
Pain pressure means (mmHg) corresponding to subjectively calibrated P10 (10-mm VAS) and P50 (50-mm VAS) represented by group (FM, HC) and TSPO polymorphism (HAB, MLAB). Error bars represent the lower and the upper 95% confidence intervals. Squares represent the mean of the data. P10, individually calibrated pain pressure matching a subjective pain rating of 10/100-mm visual analogue scale (VAS); P50, individually calibrated pain pressure matching a subjective pain rating of 50/100-mm VAS; FM, fibromyalgia; HC, healthy controls; HAB, high affinity binders; MLAB, mixed/low affinity binders; TSPO, translocator protein; VAS, visual analogue scale; FM MLAB = 30, FM HAB = 48, HC MLAB = 17, HC HAB = 21.

In the input pressure analysis, FMS showed an increased pressure pain sensitivity compared to HC, resulting in lower input pressure needed to achieve ratings corresponding to a VAS of 10 mm (P10) and 50 mm (P50) (F(1,112) = 52.89, *P* < 0.001; Fig. 3). Besides a main effect of pressure intensities (P10, P50) (F(1,112) = 658.52, *P* < 0.001; Fig. 3), a significant interaction between group and pressure intensities emerged (F(1,112) = 10.69, *P* = 0.0014; Fig. 3). No main effect of TSPO polymorphism nor significant interactions between the TSPO polymorphism and the other variables accounted for by the model were found (*P* ≥ 0.1926).

#### 3.1.4. Effects of translocator protein polymorphism and expectancy on the perceived intensity of evoked pain (P30)

**Figure 4.**
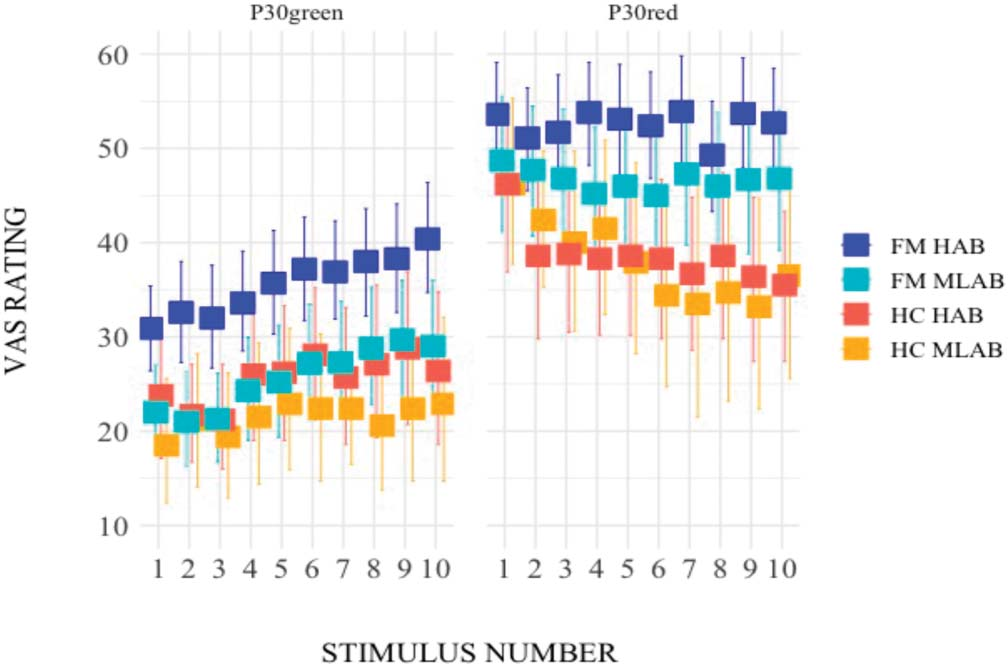
VAS pain ratings during the instructed pressure pain conditioning paradigm displayed by group (FM, HC) and TSPO polymorphism (HAB, MLAB). Error bars represent the lower and the upper 95% confidence intervals. Squares represent the mean of the data. VAS, visual analogue scale; P30green, midintensity pain pressure presented after a green cue and obtained by averaging individually calibrated low and high pain pressures, P10 (10/100-mm VAS) and P50 (50/100-mm VAS), respectively; P30red, midintensity pain pressure presented after a red cue and obtained in the same manner as P30green (see description above); FM, fibromyalgia; HAB, high affinity binders; HC, healthy controls; MLAB, mixed/low affinity binders; TSPO, translocator protein; VAS, visual analogue scale; FM MLAB = 30, FM HAB = 48, HC MLAB = 17, and HC HAB = 21.

As for the analysis of subjective pain ratings from the instructed pressure pain paradigm, all results obtained from the linear mixed-effects model can be found in Table 2. Here, pain ratings differed among the genetically inferred variants of TSPO, with individuals with TSPO HAB rating pain intensity higher than individuals with TSPO MLAB (Fig. 4). Significant differences in pain ratings were also found between cue colors, reflecting expectancy modulation of pain, with ratings being higher when the stimulus was preceded by a red cue compared to a green cue (Fig. 4). While time differences were also observed (Fig. 4), groups did not differ in subjective pain ratings per se. The observed main effects of TSPO polymorphism, cue color, and time were qualified by statistically significant interactions between TSPO polymorphism and cue color (Fig. 4), time and cue color, as well as between group and time (Fig. 4). No significant interactions between TSPO polymorphism and time, TSPO polymorphism and group, as well as between cue color and group were found.

**Table 2:** Results from the linear mixed-effects model computed on pain ratings in the instructed pressure pain conditioning paradigm.

	ß	Lower confidence limit	Upper confidence limit	t-value	*P*
TSPO	**9.426**	**2.973**	**15.880**	**2.894**	**0.0046**
Cue color	**28.649**	**24.307**	**32.991**	**12.940**	**<0.001**
Time	**1.041**	**0.474**	**1.609**	**3.598**	**<0.001**
Group	−1.361	−9.727	7.005	−0.322	0.7478
TSPO × cue color	**−4.438**	**−7.939**	**−0.938**	**−2.486**	**0.0130**
Time × cue color	**−1.281**	**−1.853**	**−0.710**	**−4.397**	**<0.001**
Group × time	**−0.696**	**−1.295**	**−0.097**	**−2.280**	**0.0227**
TSPO × time	0.070	−0.503	0.643	0.238	0.8116
TSPO × group	−4.586	−15.602	6.430	−0.825	0.4112
Cue color × group	−2.956	−6.615	0.702	−1.585	0.1132

Main effects and interactions, significant at the conventional *P* < 0.05, are presented in bold.

TSPO, translocator protein.

An interesting pattern emerged from the data, with the FM HAB group standing out and numerically providing the highest subjective pain ratings in both cue color conditions (Fig. 4). To further explore this pattern and to disentangle the directionality of the significant interaction between TSPO polymorphism and cue color, 2 post hoc repeated measures ANOVAs were performed (*P* = 0.025 to correct for multiple comparisons), separately for the green and red conditions. In the analysis looking at the effects of TSPO polymorphism, group, and time on P30green, a main effect of TSPO polymorphism (F(1,111) = 7.52, *P* = 0.0071) emerged, with individuals with TSPO HAB rating pain intensity higher than individuals with TSPO MLAB. A main effect of group (F(1,111) = 5.56, *P* = 0.0202) was also found, with FMS providing more elevated pain intensity ratings than HC. A main effect of time (F(1,111) = 38.11, *P* < 0.001) was also shown. None of the interactions reached significance (*P* ≥ 0.0798). In the analysis investigating the effects of TSPO polymorphism, group, and time on P30red, a main effect of group (F(1,111) = 9.66, *P* = 0.0024) emerged from the analysis, with FMS evaluating a midpainful stimulus following a red cue as more painful than HC. A main effect of time (F(1,111) = 5.74, *P* = 0.0182) was also found. A significant group by time interaction was observed (F(1,111) = 7.66, *P* = 0.0066), indicating that P30red ratings differed between FMS and HC over the course of the paradigm, as previously reported.^55^ However, importantly, in contrast to P30green, TSPO did not significantly influence pain ratings of midpainful stimuli after presentation of the red cue (*P* = 0.1904). None of the other interactions reached significance (*P* ≥ 0.3161).

### 3.2. Multimodal neuroimaging results

#### 3.2.1. Single-voxel ^1^H-MRS

##### 3.2.1.1. Effects of group and translocator protein polymorphism on absolute and relative glutamate and γ-aminobutyric acid concentrations in rostral anterior cingulate cortex and thalamus

Means and standard deviations (SD) of absolute and relative glutamate and GABA concentrations in rACC and thalamus are reported in Table 3. No statistically significant effects of TSPO polymorphism or group were seen for glutamate concentrations in the rACC. However, although nonsignificant, the interaction between TSPO polymorphism and group for absolute (F(1,104) = 3.89, *P* = 0.0513) as well as relative (F(1,104) = 3.11, *P* = 0.0808) glutamate concentrations in the rACC revealed a similar pattern, with higher glutamate concentrations in the rACC of FM HABs, compared to MLABs, and vice versa for HC (Table 3 for means and SD). In the thalamus, a main effect of TSPO polymorphism was shown for absolute (F(1,112) = 5.72, *P* = 0.0184), but not for relative (*P* = 0.1046), glutamate concentrations, with higher thalamic glutamate concentrations in individuals with TSPO HABs compared to MLABs (Table 3 for means and SD). No significant main effect of group nor a significant interaction between TSPO polymorphism and group were found for thalamic glutamate concentrations (*P* ≥ 0.3024). No statistically significant effects of TSPO polymorphism and/or group nor significant interactions were seen for GABA concentrations in rACC and thalamus (*P* ≥ 0.1218).

**Table 3:** Glutamate and GABA concentrations (absolute and relative) in rACC and thalamus per group (FM, HC), TSPO (HAB, MLAB), and their combination.

	N (rACC/thalamus)	rACC glutamate		THALAMUS glutamate		rACC GABA		THALAMUS GABA	
		Absolute	Relative	Absolute	Relative	Absolute	Relative	Absolute	Relative
		M (SD)	M (SD)	M (SD)	M (SD)	M (SD)	M (SD)	M (SD)	M (SD)
FM	68/74	10.8 (2.7)	10.2 (2.6)	9.3 (1.2)	9.6 (1.4)	2.4 (1.1)	2.2 (1.1)	1.9 (0.9)	2.0 (0.9)
HC	40/42	10.6 (2.4)	10.2 (1.8)	9.0 (1.3)	9.4 (1.1)	2.4 (1.0)	2.3 (0.8)	1.7 (0.8)	1.7 (0.8)
HAB	65/66	10.9 (2.7)	10.3 (2.4)	**9.4 (1.2)**	9.7 (1.4)	2.4 (1.1)	2.3 (1.1)	1.9 (0.9)	2.0 (0.9)
MLAB	43/50	10.5 (2.5)	10.0 (2.2)	8.9 (1.2)	9.3 (1.1)	2.3 (1.0)	2.2 (0.9)	1.7 (0.8)	1.8 (0.9)
FM HAB	44/45	11.3 (2.9)	10.5 (2.8)	9.5 (1.3)	9.8 (1.5)	2.4 (1.2)	2.3 (1.2)	2.1 (0.9)	2.1 (0.9)
FM MLAB	24/29	10.1 (2.3)	9.6 (2.3)	9.0 (0.9)	9.3 (1.1)	2.3 (1.0)	2.1 (0.9)	1.8 (0.8)	1.8 (0.8)
HC HAB	21/21	10.2 (2.0)	9.8 (1.3)	9.3 (0.9)	9.5 (1.2)	2.3 (1.0)	2.2 (0.9)	1.7 (0.8)	1.7 (0.8)
HC MLAB	19/21	11.0 (2.7)	10.6 (2.1)	8.7 (1.6)	9.3 (1.1)	2.4 (1.0)	2.3 (0.8)	1.6 (0.9)	1.7 (0.9)

The reported values are means (M) and standard deviations (SD) in parentheses. Absolute thalamic glutamate concentrations were significantly higher in the HAB, compared to MLAB, group (bold, *P* = 0.0184).

FM, fibromyalgia; GABA, γ-aminobutyric acid; HAB, high affinity binders; HC, healthy controls; MLAB, mixed/low affinity binders; rACC, rostral anterior cingulate cortex; TSPO, translocator protein; Absolute, absolute compound concentrations; Relative, relative compound concentrations.

##### 3.2.1.2. Correlations between glutamate and γ-aminobutyric acid concentrations in the rostral anterior cingulate cortex and thalamus

The correlations between glutamate and GABA concentrations in rACC and thalamus, respectively, are presented in Table 4. In the rACC, a consistent pattern emerged revealing significant weak to moderate positive correlations between glutamate and GABA concentrations (absolute and relative) across all subjects, in the FM, HAB, as well as in the FM HAB and HC HAB groups. No significant positive correlations were found in individuals carrying the MLAB genetic variant, in fact, a significant negative correlation was seen in the HC MLAB group for relative metabolite concentrations. Our data indicate that the HAB genetic variant is associated with positive correlations between glutamate and GABA in the rACC of FMS and HC alike. Regarding the thalamus, we found significant positive glutamate and GABA correlations (for absolute and relative concentrations) across all subjects, in the FM, HAB, and FM HAB groups. In addition, significant positive correlations were found, only for relative values, in HC, MLAB, and HC MLAB groups.

**Table 4:** Spearman correlations between glutamate and GABA concentrations (absolute and relative) in rACC and thalamus.

	N (rACC/thalamus)	rACC				THALAMUS			
		Absolute		Relative		Absolute		Relative	
		r	*P*	r	*P*	r	*P*	r	*P*
Across subjects	108/116	**0.2899**	**0.0023**	**0.2232**	**0.0202**	**0.3036**	**<0.001**	**0.3512**	**<0.001**
FM	68/74	**0.3248**	**0.0069**	**0.3541**	**0.0031**	**0.2967**	**0.0103**	**0.3461**	**0.0025**
HC	40/42	0.2387	0.1380	−0.0479	0.7694	0.3008	0.0529	**0.3381**	**0.0285**
HAB	65/66	**0.4634**	**<0.001**	**0.5289**	**<0.001**	**0.3378**	**0.0055**	**0.3090**	**0.0116**
MLAB	43/50	−0.0107	0.9460	−0.2985	0.0518	0.2122	0.1390	**0.4018**	**0.0038**
FM HAB	44/45	**0.4086**	**0.0059**	**0.5579**	**<0.001**	**0.3069**	**0.0403**	**0.3359**	**0.0241**
FM MLAB	24/29	0.0636	0.7678	−0.2039	0.3392	0.1394	0.4707	0.3303	0.0801
HC HAB	21/21	**0.5618**	**0.0080**	**0.5215**	**0.0153**	0.3355	0.1371	0.1886	0.4129
HC MLAB	19/21	−0.1053	0.6680	**−0.5035**	**0.0280**	0.2706	0.2355	**0.4518**	**0.0398**

Correlations, significant at the conventional *P* < 0.05, are presented in **bold**.

FM, fibromyalgia; HAB, high affinity binders; HC, healthy controls; MLAB, mixed/low affinity binders; rACC, rostral anterior cingulate cortex; Absolute, absolute compound concentrations; Relative, relative compound concentrations.

##### 3.2.1.3. Correlations between the conditioned pain modulation score and glutamate as well as the conditioned pain modulation score and γ-aminobutyric acid

The correlations between the CPM score, as a measure of descending pain inhibition, and absolute as well as relative glutamate and GABA concentrations in rACC and thalamus are reported in Table 5. In the rACC, a consistent pattern of positive correlations between the CPM score and absolute as well as relative glutamate or GABA concentrations emerged in the FM and the FM HAB groups, but was not seen in HC or in MLAB individuals of either group. In the thalamus, no significant correlations emerged between the CPM score and glutamate or GABA concentrations.

**Table 5:** Spearman correlations between the CPM score and glutamate as well as the CPM score and GABA in rACC and thalamus.

	N (rACC/thalamus)	rACC glutamate-CPM				THALAMUS glutamate-CPM				rACC GABA-CPM				THALAMUS GABA-CPM			
		Absolute		Relative		Absolute		Relative		Absolute		Relative		Absolute		Relative	
		r	*P*	r	*P*	r	*P*	r	*P*	r	*P*	r	*P*	r	*P*	r	*P*
Across subjects	106/113	0.1765	0.0703	**0.2093**	**0.0313**	0.0704	0.4590	0.1497	0.1134	**0.2314**	**0.0170**	**0.2413**	**0.0127**	−0.0395	0.6779	0.0082	0.9316
FM	67/72	**0.2802**	**0.0216**	**0.2754**	**0.0241**	0.0509	0.6713	0.1790	0.1324	**0.3365**	**0.0054**	**0.3329**	**0.0059**	−0.0833	0.4869	−0.0240	0.8416
HC	39/41	0.0591	0.7208	0.1644	0.3173	0.2298	0.1484	0.1991	0.2120	0.0545	0.7420	0.0289	0.8611	0.2064	0.1954	0.2041	0.2006
HAB	64/64	**0.2806**	**0.0247**	0.2255	0.0731	0.0813	0.5230	0.1335	0.2929	**0.3143**	**0.0114**	**0.2940**	**0.0184**	−0.1434	0.2584	−0.1013	0.4256
MLAB	42/49	0.0560	0.7247	0.1724	0.2751	0.1547	0.2886	0.2716	0.0590	0.1179	0.4570	0.1675	0.2889	0.1901	0.1908	0.2441	0.0910
FM HAB	44/44	**0.3951**	**0.0080**	**0.3270**	**0.0303**	0.0519	0.7381	0.1841	0.2317	**0.4208**	**0.0045**	**0.3801**	**0.0109**	−0.1592	0.3019	−0.1134	0.4636
FM MLAB	23/28	0.1206	0.5837	0.2194	0.3146	0.1445	0.4632	0.2841	0.1429	0.1573	0.4736	0.1909	0.3829	0.1768	0.3681	0.2693	0.1658
HC HAB	20/20	0.2165	0.3591	0.1504	0.5269	0.3609	0.1180	0.1805	0.4465	0.0534	0.8231	−0.0715	0.7647	0.1581	0.5055	0.1702	0.4730
HC MLAB	19/21	−0.0667	0.7863	0.0842	0.7318	0.2520	0.2706	0.4156	0.0610	0.1281	0.6013	0.1754	0.4725	0.3177	0.1606	0.3132	0.1669

Correlations, significant at the conventional *P* < 0.05, are presented in bold.

CPM, conditioned pain modulation; FM, fibromyalgia; GABA, γ-aminobutyric acid; HAB, high affinity binders; HC, healthy controls; MLAB, mixed/low affinity binders; rACC, rostral anterior cingulate cortex; Absolute, absolute compound concentrations; Relative, relative compound concentrations.

#### 3.2.2. Functional magnetic resonance imaging

First, we evaluated the main effect of pain to assess the effects of painful pressure stimulation for all P30 across the whole brain. This revealed a strong BOLD response in areas classically associated with pain processing, including insula, operculum, somatosensory cortices, and ACC (Supplementary Table 1, available at http://links.lww.com/PAIN/B362). Please see our previous study^55^ for detailed separate analyses in FMS and HC.

Based on the behavioral results, we aimed to identify cerebral differences between variants of the TSPO polymorphism in the processing of noxious stimulation. In the predetermined ROIs, ie, rACC and thalamus, there were no differences between either groups or TSPO genotypes, neither during P30green nor during P30red.

We then tested for TSPO effects whole brain, ie, in brain regions outside of the rACC and thalamus, for completion. Opposed to the TSPO by cue color interaction in the behavioral results, no pain-related difference in brain activation was observed between TSPO genetic variants depending on preceding cue. Specifically, no differences between TSPO genotypes were observed in either P30green or P30red.

In addition, no significant difference between TSPO genotypes was found in pain processing when brain response was modulated using individual pain ratings as a parametric modulator. Although VAS scores resembled more closely P10 and P50 for P30green and P30red, respectively, in the beginning of the testing phase and converged over time, this effect was not accompanied by differences in BOLD signal modulated by pain perception.

#### 3.2.3. Correlations between extracted P30 blood oxygen level-dependent signal and glutamate as well as extracted P30 blood oxygen level-dependent signal and γ-aminobutyric acid

No significant correlations between pain-evoked BOLD signal and either GABA or glutamate concentrations were observed in rACC and thalamus (Supplementary Table 2, available at http://links.lww.com/PAIN/B362).

## 4. Discussion

The translocator protein is upregulated during glial activation and increased cerebral TSPO binding has been reported in FMS.^1^ To our knowledge, the current study provides the first evidence linking the genetically inferred variants of TSPO to: (1) endogenous pain modulation, in the form of descending pain inhibition and expectancy-induced pain modulation, (2) the concentration and equilibrium of the 2 main excitatory and inhibitory neurotransmitters, ie, glutamate and GABA, and (3) the relationship between top-down pain inhibition and the above-mentioned metabolites in rACC, but not thalamus. A common denominator of these findings is that they are non–FM-specific. Individuals with genetically inferred TSPO HAB, as opposed to MLAB, demonstrated reduced descending pain inhibition and lower expectancy-induced reduction of pain, indicating less efficient endogenous pain modulation. Further supporting the role of TSPO polymorphism in pain regulation, a pattern of positive correlations emerged between conditioned pain modulation and glutamate or GABA in the rACC, a central region for pain modulation,^28^ in FMS, HABs, and FM HABs, ie, groups with reduced descending pain inhibition. Thus, in FMS an aberrant pain regulatory system combined with a HAB genetic set-up might increase the inefficiency of pain modulation. Further non–FM-specific TSPO effects presented as TSPO HABs, compared to MLABs, having higher absolute glutamate concentrations in the thalamus. In the rACC, positive correlations between glutamate and GABA were found in TSPO HABs of both groups, whereas the pattern was different in the thalamus, suggesting, speculatively, that TSPO might have brain region-specific effects on the investigated metabolites.

### 4.1. Genetically inferred translocator protein binding and endogenous pain modulation

Although TSPO did not influence clinical measures, the effects on endogenous pain modulation revealed a consistent pattern. Our CPM data are in accordance with previous findings demonstrating that FM is associated with an aberrant pain modulatory system.^31,37^ The influence of the TSPO variants on CPM revealed less efficient CPM in HABs compared to MLABs in both groups alike, indicating that CPM is affected by the TSPO polymorphism regardless of baseline pain levels and overall pain inhibition efficiency. The reduction in pain modulation efficiency in TSPO HABs becomes apparent when considering that HC with TSPO HAB took on FM-like characteristics by showing similar pain modulation scores as FMS with TSPO MLAB (Fig. 2).

Previously, we observed that FMS and HC diverged in expectancy-induced pain modulation.^55^ However, the present data show a pattern of non–FM-specific effects of TSPO, although these seemed to be more pronounced in FMS than HC (Fig. 4). The influence of TSPO was distinct during the P30green (Fig. 4), not P30red, condition. This indicates the involvement of TSPO-associated mechanisms specifically during the green condition, with TSPO HABs displaying a lower expectancy-induced reduction of pain.

### 4.2. Concentrations of glutamate and γ-aminobutyric acid in relation to the translocator protein polymorphism

No significant differences in the concentrations of glutamate or GABA were found between FMS and HC. To our knowledge, this is the first MRS study investigating the rACC in FMS, although higher glutamate concentrations in the ACC were reported in a mixed cohort of patients with chronic pain including FMS.^27^ Our results are consistent with earlier reports of similar thalamic concentrations of glutamate between FMS and HC.^12,13,59^ However, we found that absolute thalamic glutamate concentrations were higher in TSPO HABs than MLABs. Hypothetically, this finding may be related to differences in pain modulation between TSPO HABs and MLABs because glutamatergic projections from the thalamus to rACC have been implicated in pain processing.^16,22^

Of further interest is the balance between excitation and inhibition, with an altered equilibrium between glutamate and GABA being extensively proposed to contribute to several chronic pain pathologies, FM included.^50^ Here, a clear pattern of positive associations between glutamate and GABA in the rACC was observed in both FMS and HC with, uniquely, the TSPO HAB variant, whereas a negative correlation between the relative concentrations of these metabolites was seen in TSPO MLABs (although significant only in HC). Although the influence of TSPO on the relationship between thalamic glutamate and GABA seemed less clear, the displayed pattern pointed again to TSPO-related effects not being specific to FM. Altogether, our data support a brain region-specific influence of TSPO-linked mechanisms on glutamate and GABA concentrations.

Furthermore, uniquely attributable to the rACC, a significant pattern of positive correlations between glutamate and the CPM score as well as GABA and the CPM score emerged for FMS, TSPO HABs (FM and HC pooled), and FM HABs but was not significant in HC HABs. Glutamate and GABA are postulated to act in the brain as excitatory and inhibitory neurotransmitters, respectively. As such, it might seem counterintuitive that a pattern of positive correlations features, at the same time, the relationship between CPM and GABA and the one between CPM and glutamate. There are several possible explanations: (1) The MRS technique has the inherent limitation of not allowing to determine which pool of glutamate and GABA is being measured. When these neurotransmitters are in the synaptic clefts, their biological effects rely on the nature of the neurons they make synaptic contact with, as inhibition of inhibitory neurons (GABA) or excitation of excitatory neurons (glutamate) could give rise to similar biological effects,^14^ (2) in the context of the glutamine-glutamate/GABA cycle,^61^ both GABA and glutamate potentially have both pronociceptive and antinociceptive effects based on their concentrations, site, and type of receptors that is activated,^43,49^ and (3) correlations do not reveal causality. Our results suggest a functional link between TSPO polymorphism, CPM, as well as the glutamate and GABA equilibrium in the rACC.

### 4.3. Cerebral pain-related activation in relation to magnetic resonance spectroscopy data and the translocator protein polymorphism

In this study, glutamate and GABA in rACC and thalamus were not found to be associated with neural activity in these brain regions during evoked pain. In the FM literature, some studies have reported an association between baseline glutamate and changes in cerebral response to evoked pain in the insula^20,21^ while, to our knowledge, none has been published regarding GABA. Due to the documented involvement of the rACC and thalamus in FM^28–30^ and the notion that the projections from the thalamus to ACC are glutamatergic, while ACC neurons respond to thalamic input via GABAergic-mediated inhibition,^16^ we explored whether GABA and glutamate in rACC and thalamus were related to cerebral pain processing. One explanation for the lack of association between task-related fMRI BOLD signal and MRS during rest might reside in the fact that data were collected at different time points, suggesting the need for both techniques to be task-based. Furthermore, despite the behavioral results, we found no evidence of the effect of the TSPO variants on cerebral pain processing in BOLD response, which is in accordance with our previous findings.^38^ The BOLD signal relies on neurovascular coupling during neuronal activation resulting from the release of excitatory neurotransmitter substances, such as glutamate.^42^ Contrary to this, GABA, as an inhibitory transmitter, has been proposed to be a key mediator of negative BOLD responses, also referred to as deactivations, mainly related to inhibitory postsynaptic potentials.^4,25,44^ Hypothetically, a simultaneous release of glutamate and GABA in the same neuronal cluster could have opposing effects on the neurovascular response and, in turn, affect the BOLD signal. Therefore, the equilibrium between glutamate and GABA release in a brain region would have physiological effects not necessarily captured by the BOLD response. Under this assumption, our findings showed that the TSPO polymorphism was associated with pain modulation on the behavioral level and the equilibrium between glutamate and GABA, particularly in the rACC, although without detectable differences in pain-related BOLD signal.

### 4.4. Limitations

First, due to uneven group sizes, some statistical analyses were less powered than possible with equal-sized groups. Second, MRS data were acquired at rest, reflecting baseline metabolite concentrations, thus questioning direct relation to task-based fMRI. Third, standard PRESS, the most commonly used sequence in clinical studies, may not be the optimal method to separate glutamate, glutamine, and GABA. Higher accuracy in metabolite detection could be achieved via a semi-LASER sequence, as this has been recently suggested to reduce PRESS-related localization errors.^48^

## 5. Conclusions

In FMS and HC alike, the TSPO HAB variant was associated with a reduced efficacy of endogenous pain modulation, ie, less efficient descending pain inhibition and diminished expectancy-induced reduction of pain. Further supporting the TSPO involvement in pain regulation were the positive associations between conditioned pain modulation and glutamate or GABA in the rACC, a central region for pain modulation,^28^ in FMS, HABs, and FM HABs, ie, groups with reduced descending pain inhibition. Moreover, in the rACC of HABs, but not MLABs of both groups, a pattern of positive correlations between glutamate and GABA was found, whereas there was no influence of TSPO polymorphism on the concentration of such metabolites. However, as HABs of both groups had higher absolute thalamic glutamate concentrations than MLABs, our findings point to TSPO-linked effects being brain region-specific. Altogether, our data indicate an important non–FM-specific role of TSPO in the regulation of endogenous pain modulation and brain metabolism, thereby supporting the drug development targeting TSPO-related mechanisms for pain relief.

## Conflict of interest statement

The authors have no conflicts of interest to declare.

## Appendix A. Supplemental digital content

Supplemental digital content associated with this article can be found online at http://links.lww.com/PAIN/B362.

## Supplementary Material

SUPPLEMENTARY MATERIAL

## References

[1] Albrecht DS Forsberg A Sandström A Bergan C Kadetoff D Protsenko E Lampa J Lee YC Höglund CO Catana C Cervenka S Akeju O Lekander M Cohen G Halldin C Taylor N Kim M Hooker JM Edwards RR Napadow V Kosek E Loggia ML. Brain glial activation in fibromyalgia—a multi-site positron emission tomography investigation. Brain Behav Immun 2019;75:72–83. 30223011 10.1016/j.bbi.2018.09.018 PMC6541932

[2] Bäckryd E Tanum L Lind AL Larsson A Gordh T. Evidence of both systemic inflammation and neuroinflammation in fibromyalgia patients, as assessed by a multiplex protein panel applied to the cerebrospinal fluid and to plasma. J Pain Res 2017;10:515–25. 28424559 10.2147/JPR.S128508 PMC5344444

[3] Bennett RM Jones J Turk DC Russell IJ Matallana L. An internet survey of 2,596 people with fibromyalgia. BMC Musculoskelet Disord 2007;8:27. 17349056 10.1186/1471-2474-8-27 PMC1829161

[4] Boorman L Kennerley AJ Johnston D Jones M Zheng Y Redgrave P Berwick J. Negative blood oxygen level dependence in the rat:A model for investigating the role of suppression in neurovascular coupling. J Neurosci 2010;30:4285–94. 20335464 10.1523/JNEUROSCI.6063-09.2010 PMC6634501

[5] Burckhardt CS Clark SR Bennett RM. The fibromyalgia impact questionnaire: development and validation. J Rheumatol 1991;18:728–33. 1865419

[6] Clauw DJ. Fibromyalgia: a clinical review. JAMA 2014;311:1547. 24737367 10.1001/jama.2014.3266

[7] Contopoulos-Ioannidis DG Karvouni A Kouri I Ioannidis JPA. Reporting and interpretation of SF-36 outcomes in randomised trials: systematic review. BMJ 2009;338:a3006. 19139138 10.1136/bmj.a3006 PMC2628302

[8] Costa B Da Pozzo E Martini C. Translocator protein as a promising target for novel anxiolytics. Curr Top Med Chem 2012;12:270–85. 22204481 10.2174/156802612799078720

[9] Crawshaw AA Robertson NP. The role of TSPO PET in assessing neuroinflammation. J Neurol 2017;264:1825–7. 28698896 10.1007/s00415-017-8565-1 PMC5533836

[10] Da Pozzo E Costa B Martini C. Translocator protein (TSPO) and neurosteroids: implications in psychiatric disorders. Curr Mol Med 2012;12:426–42. 22348611 10.2174/156652412800163451

[11] Fayed N Andres E Rojas G Moreno S Serrano-Blanco A Roca M Garcia-Campayo J. Brain dysfunction in fibromyalgia and somatization disorder using proton magnetic resonance spectroscopy: a controlled study: brain dysfunction in somatization. Acta Psychiatr Scand 2012;126:115–25. 22211322 10.1111/j.1600-0447.2011.01820.x

[12] Fayed N Garcia-Campayo J Magallón R Andrés-Bergareche H Luciano JV Andres E Beltrán J. Localized 1H-NMR spectroscopy in patients with fibromyalgia: a controlled study of changes in cerebral glutamate/glutamine, inositol, choline, and N-acetylaspartate. Arthritis Res Ther 2010;12:R134. 20609227 10.1186/ar3072 PMC2945024

[13] Feraco P Bacci A Pedrabissi F Passamonti L Zampogna G Pedrabissi Fed Malavolta N Leonardi M. Metabolic abnormalities in pain-processing regions of patients with fibromyalgia: a 3T MR spectroscopy study. Am J Neuroradiol 2011;32:1585–90. 21799042 10.3174/ajnr.A2550 PMC7965402

[14] Fields H. State-dependent opioid control of pain. Nat Rev Neurosci 2004;5:565–75. 15208698 10.1038/nrn1431

[15] Foerster BR Petrou M Edden RAE Sundgren PC Schmidt-Wilcke T Lowe SE Harte SE Clauw DJ Harris RE. Reduced insular γ-aminobutyric acid in fibromyalgia. Arthritis Rheum 2012;64:579–83. 21913179 10.1002/art.33339 PMC3374930

[16] Gigg J Tan AM Finch DM. Glutamatergic excitatory responses of anterior cingulate neurons to stimulation of the mediodorsal thalamus and their regulation by GABA: an in vivo lontophoretic study. Cereb Cortex 1992;2:477–84. 1282403 10.1093/cercor/2.6.477

[17] Govind V Young K Maudsley AA. Corrigendum: proton NMR chemical shifts and coupling constants for brain metabolites. Govindaraju V, Young K, Maudsley AA, NMR Biomed. 2000;13:129–153. NMR Biomed 2015;28:923–4. 26094860 10.1002/nbm.3336

[18] Govindaraju V Young K Maudsley AA. Proton NMR chemical shifts and coupling constants for brain metabolites. NMR Biomed 2000;13:129–53. 10861994 10.1002/1099-1492(200005)13:3<129::aid-nbm619>3.0.co;2-v

[19] Harris RE. Elevated excitatory neurotransmitter levels in the fibromyalgia brain. Arthritis Res Ther 2010;12:141. 20959024 10.1186/ar3136 PMC2991003

[20] Harris RE Sundgren PC Craig AD Kirshenbaum E Sen A Napadow V Clauw DJ. Elevated insular glutamate in fibromyalgia is associated with experimental pain. Arthritis Rheum 2009;60:3146–52. 19790053 10.1002/art.24849 PMC2827610

[21] Harris RE Sundgren PC Pang Y Hsu M Petrou M Kim S-H McLean SA Gracely RH Clauw DJ. Dynamic levels of glutamate within the insula are associated with improvements in multiple pain domains in fibromyalgia. Arthritis Rheum 2008;58:903–7. 18311814 10.1002/art.23223

[22] Harte SE Spuz CA Borszcz GS. Functional interaction between medial thalamus and rostral anterior cingulate cortex in the suppression of pain affect. Neuroscience 2011;172:460–73. 21034797 10.1016/j.neuroscience.2010.10.055 PMC3030197

[23] Häuser W Brähler E Ablin J Wolfe F. 2016 modified American College of Rheumatology fibromyalgia criteria, ACTTION-APS Pain Taxonomy criteria and the prevalence of fibromyalgia. Arthritis Care Res 2020. doi: 10.1002/acr.24202 [Epub ahead of print]. 32248629

[24] Hawker GA Mian S Kendzerska T French M. Measures of adult pain: visual analog scale for pain (VAS pain), numeric rating scale for pain (NRS pain), McGill pain questionnaire (MPQ), short-form McGill pain questionnaire (SF-MPQ), chronic pain grade scale (CPGS), short form-36 bodily pain scale (SF-36 BPS), and measure of intermittent and constant osteoarthritis pain (ICOAP). Arthritis Care Res 2011;63:S240–52. 10.1002/acr.20543 22588748

[25] Hayes DJ Huxtable AG. Interpreting deactivations in neuroimaging. Front Psychol 2012;3:27. 22347207 10.3389/fpsyg.2012.00027 PMC3273719

[26] Henn FA Goldstein MN Hamberger A. Uptake of the neurotransmitter candidate glutamate by glia. Nature 1974;249:663–4. 4365397 10.1038/249663a0

[27] Ito T Tanaka-Mizuno S Iwashita N Tooyama I Shiino A Miura K Fukui S. Proton magnetic resonance spectroscopy assessment of metabolite status of the anterior cingulate cortex in chronic pain patients and healthy controls. J Pain Res 2017;10:287–93. 28203104 10.2147/JPR.S123403 PMC5293371

[28] Jensen KB Kosek E Petzke F Carville S Fransson P Marcus H Williams SCR Choy E Giesecke T Mainguy Y Gracely R Ingvar M. Evidence of dysfunctional pain inhibition in Fibromyalgia reflected in rACC during provoked pain. PAIN 2009;144:95–100. 19410366 10.1016/j.pain.2009.03.018

[29] Jensen KB Loitoile R Kosek E Petzke F Carville S Fransson P Marcus H Williams SC Choy E Mainguy Y Vitton O Gracely RH Gollub R Ingvar M Kong J. Patients with fibromyalgia display less functional connectivity in the brain's pain inhibitory network. Mol Pain 2012;8:32. 22537768 10.1186/1744-8069-8-32 PMC3404927

[30] Jensen KB Srinivasan P Spaeth R Tan Y Kosek E Petzke F Carville S Fransson P Marcus H Williams SCR Choy E Vitton O Gracely R Ingvar M Kong J. Overlapping structural and functional brain changes in patients with long-term exposure to fibromyalgia pain: brain changes in long-term fibromyalgia. Arthritis Rheum 2013;65:3293–303. 23982850 10.1002/art.38170 PMC3984030

[31] Julien N Goffaux P Arsenault P Marchand S. Widespread pain in fibromyalgia is related to a deficit of endogenous pain inhibition. PAIN 2005;114:295–302. 15733656 10.1016/j.pain.2004.12.032

[32] Kadetoff D Lampa J Westman M Andersson M Kosek E. Evidence of central inflammation in fibromyalgia—increased cerebrospinal fluid interleukin-8 levels. J Neuroimmunol 2012;242:33–8. 22126705 10.1016/j.jneuroim.2011.10.013

[33] Kantamneni S. Cross-talk and regulation between glutamate and GABAB receptors. Front Cell Neurosci 2015;9:135. 25914625 10.3389/fncel.2015.00135 PMC4392697

[34] Kong J Jensen K Loiotile R Cheetham A Wey HY Tan Y Rosen B Smoller JW Kaptchuk TJ Gollub RL. Functional connectivity of the frontoparietal network predicts cognitive modulation of pain. PAIN 2013;154:459–67. 23352757 10.1016/j.pain.2012.12.004 PMC3725961

[35] Kosek E Altawil R Kadetoff D Finn A Westman M Le Maître E Andersson M Jensen-Urstad M Lampa J. Evidence of different mediators of central inflammation in dysfunctional and inflammatory pain—interleukin-8 in fibromyalgia and interleukin-1 β in rheumatoid arthritis. J Neuroimmunol 2015;280:49–55. 25773155 10.1016/j.jneuroim.2015.02.002 PMC4372266

[36] Kosek E Cohen M Baron R Gebhart GF Mico J-A Rice ASC Rief W Sluka AK. Do we need a third mechanistic descriptor for chronic pain states?. PAIN 2016;157:1382–6. 26835783 10.1097/j.pain.0000000000000507

[37] Kosek E Hansson P. Modulatory influence on somatosensory perception from vibration and heterotopic noxious conditioning stimulation (HNCS) in fibromyalgia patients and healthy subjects. PAIN 1997;70:41–51. 9106808 10.1016/s0304-3959(96)03295-2

[38] Kosek E Martinsen S Gerdle B Mannerkorpi K Löfgren M Bileviciute-Ljungar I Fransson P Schalling M Ingvar M Ernberg M Jensen KB. The translocator protein gene is associated with symptom severity and cerebral pain processing in fibromyalgia. Brain Behav Immun 2016;58:218–27. 27448744 10.1016/j.bbi.2016.07.150

[39] Lavisse S Guillermier M Herard AS Petit F Delahaye M Van Camp N Ben Haim L Lebon V Remy P Dolle F Delzescaux T Bonvento G Hantraye P Escartin C. Reactive astrocytes overexpress TSPO and are detected by TSPO positron emission tomography imaging. J Neurosci 2012;32:10809–18. 22875916 10.1523/JNEUROSCI.1487-12.2012 PMC6621018

[40] Lindstedt F Berrebi J Greayer E Lonsdorf TB Schalling M Ingvar M Kosek E. Conditioned pain modulation is associated with common polymorphisms in the serotonin transporter gene. PLoS One 2011;6:e18252. 21464942 10.1371/journal.pone.0018252 PMC3065474

[41] Loggia ML Berna C Kim J Cahalan CM Gollub RL Wasan AD Harris RE Edwards RR Napadow V. Disrupted brain circuitry for pain-related reward/punishment in fibromyalgia: reward/punishment brain circuitry in fibromyalgia. Arthritis Rheumatol 2014;66:203–12. 24449585 10.1002/art.38191 PMC4516215

[42] Magistretti PJ Pellerin L. Cellular mechanisms of brain energy metabolism and their relevance to functional brain imaging. Philos Trans R Soc Lond B Biol Sci 1999;354:1155–63. 10466143 10.1098/rstb.1999.0471 PMC1692634

[43] McCarson KE Enna SJ. GABA pharmacology: the search for analgesics. Neurochem Res 2014;39:1948–63. 24532294 10.1007/s11064-014-1254-x

[44] Muthukumaraswamy SD Evans CJ Edden RAE Wise RG Singh KD. Individual variability in the shape and amplitude of the BOLD-HRF correlates with endogenous GABAergic inhibition. Hum Brain Mapp 2012;33:455–65. 21416560 10.1002/hbm.21223 PMC3374935

[45] Nothdurfter C Baghai TC Schüle C Rupprecht R. Translocator protein (18 kDa) (TSPO) as a therapeutic target for anxiety and neurologic disorders. Eur Arch Psychiatry Clin Neurosci 2012;262:107–12. 22923187 10.1007/s00406-012-0352-5

[46] O'Brien AT Deitos A Triñanes Pego Y Fregni F Carrillo-de-la-Peña MT. Defective endogenous pain modulation in fibromyalgia: a meta-analysis of temporal summation and conditioned pain modulation paradigms. J Pain 2018;19:819–36. 29454976 10.1016/j.jpain.2018.01.010

[47] Owen DR Yeo AJ Gunn RN Song K Wadsworth G Lewis A Rhodes C Pulford DJ Bennacef I Parker CA StJean PL Cardon LR Mooser VE Matthews PM Rabiner EA Rubio JP. An 18-kDa translocator protein (TSPO) polymorphism explains differences in binding affinity of the PET radioligand PBR28. J Cereb Blood Flow Metab 2012;32:1–5. 22008728 10.1038/jcbfm.2011.147 PMC3323305

[48] Öz G Deelchand DK Wijnen JP Mlynárik V Xin L Mekle R Noeske R Scheenen TWJ Tkáč I, the Experts' Working Group on Advanced Single Voxel H MRS, Andronesi O Barker PB Bartha R Berrington A Boer V Cudalbu C Emir UE Ernst T Fillmer A Heerschap A Henry P Hurd RE Joers JM Juchem C Kan HE Klomp DWJ Kreis R Landheer K Mangia S Marjańska M Near J Ratai EM Ronen I Slotboom J Soher BJ Terpstra M Valette J Van der Graaf M Wilson M. Advanced single voxel 1H magnetic resonance spectroscopy techniques in humans: experts' consensus recommendations. NMR Biomed 2021;34:e4236. 10.1002/nbm.4236 PMC7347431 31922301

[49] Palazzo E de Novellis V Rossi F Maione S. Supraspinal metabotropic glutamate receptor subtype 8: a switch to turn off pain. Amino Acids 2014;46:1441–8. 24623118 10.1007/s00726-014-1703-5

[50] Peek AL Rebbeck T Puts NAJ Watson J Aguila MER Leaver AM. Brain GABA and glutamate levels across pain conditions: a systematic literature review and meta-analysis of 1H-MRS studies using the MRS-Q quality assessment tool. NeuroImage 2020;210:116532. 31958584 10.1016/j.neuroimage.2020.116532

[51] Pinhero J Bates D DebRoy S Sarkar D; R Core Team. Nlme: linear and nonlinear mixed effects models 2018. Available at: https://CRAN.R-project.org/package=nlme. Accessed February 18, 2020.

[52] Power JD Barnes KA Snyder AZ Schlaggar BL Petersen SE. Spurious but systematic correlations in functional connectivity MRI networks arise from subject motion. NeuroImage 2012;59:2142–54. 22019881 10.1016/j.neuroimage.2011.10.018 PMC3254728

[53] RStudioTeam. RStudio: Integrated Development Environment for R. Boston, MA, 2016. Available at: http://www.rstudio.com/. Accessed February 18, 2020.

[54] Rupprecht R Papadopoulos V Rammes G Baghai TC Fan J Akula N Groyer G Adams D Schumacher M. Translocator protein (18 kDa) (TSPO) as a therapeutic target for neurological and psychiatric disorders. Nat Rev Drug Discov 2010;9:971–88. 21119734 10.1038/nrd3295

[55] Sandström A Ellerbrock I Tour J Kadetoff D Jensen KB Kosek E. Neural correlates of conditioned pain responses in fibromyalgia subjects indicate preferential formation of new pain associations rather than extinction of irrelevant ones. PAIN 2020;161:2079–88. 32379218 10.1097/j.pain.0000000000001907 PMC7431138

[56] Sluka KA Clauw DJ. Neurobiology of fibromyalgia and chronic widespread pain. Neuroscience 2016;338:114–29. 27291641 10.1016/j.neuroscience.2016.06.006 PMC5083139

[57] Sullivan MJL Bishop SR Pivik J. The pain catastrophizing scale: development and validation. Psychol Assess 1995;7:524–32.

[58] Tour J Löfgren M Mannerkorpi K Gerdle B Larsson A Palstam A Bileviciute-Ljungar I Bjersing J Martin I Ernberg M Schalling M Kosek E. Gene-to-gene interactions regulate endogenous pain modulation in fibromyalgia patients and healthy controls—antagonistic effects between opioid and serotonin-related genes. PAIN 2017;158:1194–203. 28282362 10.1097/j.pain.0000000000000896 PMC5472004

[59] Valdés M Collado A Bargalló N Vázquez M Rami L Gómez E Salamero M. Increased glutamate/glutamine compounds in the brains of patients with fibromyalgia: a magnetic resonance spectroscopy study. Arthritis Rheum 2010;62:1829–36. 20191578 10.1002/art.27430

[60] Wager TD Atlas LY. The neuroscience of placebo effects: connecting context, learning and health. Nat Rev Neurosci 2015;16:403–18. 26087681 10.1038/nrn3976 PMC6013051

[61] Walls AB Waagepetersen HS Bak LK Schousboe A Sonnewald U. The glutamine–glutamate/GABA cycle: function, regional differences in glutamate and GABA production and effects of interference with GABA metabolism. Neurochem Res 2015;40:402–9. 25380696 10.1007/s11064-014-1473-1

[62] Werry EL Bright FM Piguet O Ittner LM Halliday GM Hodges JR Kiernan MC Loy CT Kril JJ Kassiou M. Recent developments in TSPO PET imaging as A biomarker of neuroinflammation in neurodegenerative disorders. Int J Mol Sci 2019;20:3161. 10.3390/ijms20133161 PMC6650818 31261683

[63] Wolfe F Ross K Anderson J Russell IJ Hebert L. The prevalence and characteristics of fibromyalgia in the general population: fibromyalgia prevalence and characteristics. Arthritis Rheum 1995;38:19–28. 7818567 10.1002/art.1780380104

[64] Zigmond AS Snaith RP. The hospital anxiety and depression scale. Acta Psychiatr Scand 1983;67:361–70. 6880820 10.1111/j.1600-0447.1983.tb09716.x

