# Supplementary material for: The translocator protein gene is associated with endogenous pain modulation and the balance between glutamate and γ-aminobutyric acid in fibromyalgia and healthy subjects: a multimodal neuroimaging study
Source: Pain. 2021 Apr 9;163(2):274–86. doi: 10.1097/j.pain.0000000000002309 (PMC8756347; doi:10.1097/j.pain.0000000000002309)
Supplement: SUPPLEMENTARY MATERIAL [file jop-163-0274-s001.pdf]

Supplementary Table 1.

Site	size	t-value	FWE	x	y	z
**R Parietal Operculum**	3262	20.77	< 0.001	54	-28	22
**R Central Operculum**		15.15		56	2	4
**R Insular Cortex**		13.80		34	-20	14
**R Postcentral Gyrus**	1120	16.60	< 0.001	12	-38	68
**R Precentral Gyrus**		12.64		4	-14	64
**L Parietal Operculum**	2228	14.57	< 0.001	-52	-34	20
**L Central Operculum**		12.58		-52	-2	2
**L Postcentral Gyrus/Supramarginal Gyrus**		12.29		-62	-24	24
**R Anterior Cingulate Cortex**	403	8.19	< 0.001	6	-8	42
**L Paracingulate Gyrus**		7.14		-6	12	46
**R Anterior Cingulate Cortex**		6.29		4	2	44
**R Putamen**	31	6.35	0.001	20	12	-8

Anatomical site, cluster size (minimum size of 10 voxels), maximum t-value, p-values per cluster corrected for multiple comparisons using a family-wise error (FWE) rate approach ($p < 0.05$) and Montreal Neurological Institute-coordinates of the local maxima for the main effect of P30 pooled across subjects. L, left; R, right.

Supplementary Table 2. Spearman's correlations between P30 BOLD signal and glutamate as well as between P30 BOLD signal and GABA in rACC and thalamus.

		rACC glutamate - BOLD				THALAMUS glutamate - BOLD				rACC GABA - BOLD				THALAMUS GABA - BOLD			
		Absolute compound concentrations		Relative compound concentrations		Absolute compound concentrations		Relative compound concentrations		Absolute compound concentrations		Relative compound concentrations		Absolute compound concentrations		Relative compound concentrations	
	N (rACC/thalamus)	r	p-value	r	p-value	r	p-value	r	p-value	r	p-value	r	p-value	r	p-value	r	p-value
ACROSS SUBJECTS	94/98	-0.0796	0.4460	-0.0092	0.9299	-0.0872	0.3933	-0.0116	0.9096	0.0698	0.5041	0.0810	0.4376	-0.0669	0.5129	-0.0464	0.6500
FM	61/64	-0.2491	0.0531	-0.1574	0.2250	-0.1099	0.3864	0.0903	0.4768	0.1455	0.2631	0.1772	0.1719	-0.0008	0.9951	0.0027	0.9831
HC	33/34	0.1654	0.3560	0.2814	0.1126	-0.0918	0.6042	0.1545	0.3816	-0.0003	0.9985	-0.0010	0.9956	-0.2404	0.1709	-0.1691	0.3389
HAB	55/55	-0.0203	0.8830	0.0742	0.5891	-0.1670	0.2231	-0.0716	0.6036	0.1029	0.4549	0.1305	0.3423	-0.1633	0.2336	-0.1758	0.2155
MLAB	39/43	-0.1630	0.3204	-0.1180	0.4729	-0.0190	0.9036	0.0616	0.6938	0.0009	0.9956	-0.0078	0.9624	0.0743	0.6358	0.0947	0.5457

FM, fibromyalgia; HC, healthy controls; HAB, high affinity binders; MLAB, mixed/low affinity binders; rACC, rostral anterior cingulate cortex; BOLD, blood oxygen level-dependent; GABA, γ-aminobutyric acid.
